# Buffalo Academy of Medicine

**Published:** 1903-06

**Authors:** William Irving Thornton


### SOCIETY PROCEEDINGS.

### Buffalo Academy of Medicine.

*Section on Medicine, May 13, 1903.*

Reported by WILLIAM IRVING THORNTON, M. D.

The medical section of the Buffalo Academy of Medicine held its regular meeting in the Academy rooms, Buffalo Public Library Building, on Wednesday evening, May 13. The meeting was called to order by Dr. Norman L. Burnham, who presided in the absence of the regular chairman.

Dr. Charles S. Jewett presented a paper entitled The medical and surgical treatment of Bright's disease. He gave an outline of the medical treatment of the several varieties of nephritis and discussed Edebohl's operation of decapsulation of the kidney. A report of the first recorded autopsy after Edebohl's operation was given.

Dr. P. W. Van Peyma congratulated the essayist upon his study of the subject.

Dr. J. Henry Dowd reported a case of Edebohl's operation. The result was favorable.

Dr. G. A. Himmelsbach urged the use of normal salt solution, subcutaneously or intravenously, to combat the toxemia by hastening elimination. He cited a case where from almost total suppression a patient was made to pass 32 pints of urine in 72 hours, after injecting subcutaneously one pint of normal salt solution.

The meeting adjourned at 9.45. Number of Fellows present, 26.

